# Factors associated with screening positive for high falls risk in fragility fracture patients: a cross-sectional study

**DOI:** 10.1186/s12891-020-03410-2

**Published:** 2020-06-12

**Authors:** Nooshin K. Rotondi, Dorcas E. Beaton, Rebeka Sujic, Earl Bogoch, Taucha Inrig, Denise Linton, Jennifer Weldon, Ravi Jain, Joanna E. M. Sale

**Affiliations:** 1 Faculty of Health Sciences, Ontario Tech University, 2000 Simcoe St. North, Oshawa, Ontario L1H 7K4 Canada; 2 Musculoskeletal Health & Outcomes Research, Li Ka Shing Knowledge Institute, St. Michael’s Hospital, Toronto, Ontario Canada; 3 Institute of Health Policy, Management & Evaluation, University of Toronto, Toronto, Ontario Canada; 4 Institute for Work & Health, University of Toronto, Toronto, Ontario Canada; 5 Mobility Program, St. Michael’s Hospital, Toronto, Ontario Canada; 6 Department of Surgery, University of Toronto, Toronto, Ontario Canada; 7 Osteoporosis Canada, Toronto, Ontario Canada

**Keywords:** Fragility fracture, Risk of falling, STEADI (Stopping Elderly Accidents, Deaths & Injuries), Cross-sectional observational study

## Abstract

**Background:**

We sought to report the prevalence of fragility fracture patients who were screened at high falls risk using a large provincial database, and to determine the characteristics associated with being screened at high falls risk.

**Methods:**

The study population included fragility fracture patients 50+ years of age who were screened at 35 hospital fracture clinics in Ontario over a 3.5 year period. The outcome was based on two screening questions measuring the risk of falling, both adapted from the STEADI (Stopping Elderly Accidents, Deaths & Injuries) tool. Multivariable associations of sociodemographic, fracture-related, and health-related characteristics were evaluated using logistic regression.

**Results:**

Of the sample, 9735 (44.5%) patients were classified as being at high falls risk, and 12,089 (55.3%) were not. In the multivariable logistic regression, being 80+ years of age (vs. 50–64 years of age), non-community dwelling (vs. living with spouse, family member, roommate), having a mental/physical impairment (vs. none), and taking multiple medications, were all strongly associated with being screened at high falls risk.

**Conclusions:**

Living in a non-community dwelling and taking 4+ medications were the variables most strongly associated with being screened at high falls risk. These are potentially modifiable characteristics that should be considered when assessing falls risk in fragility fracture patients, and particularly when designing interventions for preventing subsequent falls. Ongoing work to address the higher risk of falls in the fragility fracture population is warranted.

## Background

Falls among older adults are a common and serious problem, leading to potentially severe injuries such as fractures [1–3] and head injuries [2, 3]. People over 65 years of age have the highest risk of falling, with nearly one-quarter to one-third living in the community falling at least once per year [2, 4, 5]. Older adults with osteoporosis are particularly vulnerable to sustaining a fracture from low impact forces such as falling from a standing height, known as a “fragility fracture” [2, 6, 7]. Previous studies have shown that patients with a prevalent fragility fracture have a high risk of subsequent falls [1, 8] and recurrent fractures [1, 9] and there is a strong association between falls, fall-related fractures, and functional decline [9–11]. Recurrent fractures within the fragility fracture population are particularly worrisome as they can result in severe morbidity and an increased mortality risk [12].

Research into the epidemiology of falls in older adults has identified a number of risk factors for falling, including advanced age [5, 13, 14], female gender [2, 15], chronic disease burden [4, 16], the use of multiple prescribed medications [17, 18], irrespective of type [19–22], impaired balance and gait [1, 3, 20, 23], living situation/conditions (e.g., residential care, community dwellers) [3, 7, 24]; high alcohol intake [2, 3, 7] history of previous falls [1, 3, 14], low Vitamin D intake [4, 7, 25, 26], impaired cognition [3, 7, 20, 21]; and low body weight [27].

The majority of studies examining the risk factors for falling are based on older people living in the community; however, studies of falls in participants with prevalent fragility fractures are relatively scarce despite their high risk for falls and recurrent fractures [1, 9]. In Ontario, Canada, a system-wide Fracture Screening and Prevention Program (FSPP) was established in 2007 [28] to improve the care of people who have had a fragility fracture and to facilitate interventions with the goal of preventing further fractures. About 8000 fragility fracture patients are screened and enrolled in the FSPP each year. Understanding the factors associated with falls in this population is essential to the design of appropriate fall-prevention and treatment strategies. This is an important goal because falling is a condition that should be amenable to prevention, given that there is evidence of preventability, a high rate of morbidity, and high frequency [26], although there are contradictory findings regarding whether falls prevention initiatives reduce injuries or fractures [29]. The purpose of the present study was to examine possible associated factors for being screened at high falls risk in a large fragility fracture population using a provincial database. Specifically, we sought to report the prevalence of fragility fracture patients enrolled in the FSPP who were screened at high falls risk, and to determine the characteristics associated with being screened at high falls risk.

## Methods

The study population consisted of fragility fracture patients 50 years of age or older who were screened by Fracture Prevention Coordinators (FPCs) at 35 hospital fracture clinics over a 3.5 year period. To identify individuals with a fragility fracture, FPCs asked each patient how they broke their bone(s). In our Fracture Screening and Prevention Program, we consider a fragility fracture as one that occurs as a result of a fall from standing height or less [30]. We also recognize that some fractures, such as vertebral fractures, occur from no trauma at all, e.g., just bending over to pick something up. Examples of low trauma, or fragility fractures include: slip and fall on snow, ice, or a wet floor; fall out of bed or out of a chair. The FPCs facilitate guideline implementation with respect to care management after a fragility fracture; furthermore, they collect baseline and follow-up survey data for quality assurance purposes on a routine basis. As part of usual care, the FPCs collect patient data using standardized survey instruments on tablet computers, and the data are uploaded to a central repository. The approval for use of the de-identified quality assurance data for research purposes was provided by the Research Ethics Board at St. Michael’s Hospital in Toronto, Ontario (REB# 13–156).

### Measures

#### Outcome

The outcome was based on two screening questions measuring the risk of falling. The first question asked “Have you fallen in the past year besides the fall that may have led to the current fracture(s)?”, and the second question asked “Do you have trouble getting out of a chair or feel unsteady when you walk?” These were adapted from two of three screening items in the STEADI (Stopping Elderly Accidents, Deaths & Injuries) tool from the Centers for Disease Control and Prevention [31]. Patients were considered at high falls risk if they answered “Yes” to either of these screening questions.

#### Sociodemographic and fracture-related characteristics

Demographic variables included age (categorized as 50–64, 65–79, 80+) and sex (male/female). Living arrangement was grouped into three categories: alone in house or apartment; with spouse, family member or roommate; non-community dwelling (nursing home, other residential facility or other living situation). Fracture location was grouped into five categories; hip, shoulder, wrist, and ankle represent the most commonly reported fracture types, while ‘other’ includes all other fractures which occurred too infrequently to be included in their own categories.

#### Health-related characteristics

Patients were asked if they had broken another bone since the age of 40 from a simple trip and fall, as a proxy measure for having a history of previous falls resulting in fracture. Alcohol use (drinking 3 or more alcoholic beverages a day) and vitamin D supplementation (yes/no) were also assessed. Fracture Prevention Coordinators also determined whether or not patients had any form of impairment or barrier to completing the questionnaire, including language barrier, or mental/physical impairments. The latter served as a proxy for impaired cognition because we did not specifically measure cognitive deficit in our survey. Low body weight (< 60 kg) [9] was assessed by asking patients to self-report their weight.

Patients were also asked to report if they had ever been told they had any of the following conditions: diabetes, arthritis, hearth disease (stroke, heart attack), high blood pressure, cholesterol, respiratory disease (asthma, emphysema, COPD), and cancer. A chronic disease burden variable was created by counting the number of conditions reported by each patient. The variable was categorized into four groups: 3 or more conditions, 2 conditions, 1 condition, and no condition. For each condition selected, patients were then asked to self-report if they were currently taking any medication for that condition. A variable was created counting the number of medications reported by each patient, irrespective of type, and was categorized into five groups: 4 or more medications, 3, 2, 1, and no medications. The last category included patients who were not diagnosed with any conditions and were therefore not taking any medications, in addition to those who were diagnosed with at least one condition but were not taking medications.

### Analysis

#### Description of sample

Descriptive statistics including frequencies and proportions were calculated for the outcome variable and characteristics or covariates of interest.

#### Factors associated with being screened at high falls risk

Unadjusted associations between the outcome and variables of interest were examined using logistic regression. A trend test evaluating the association between count of current medications and the outcome was also performed. For this test, the former variable was coded as a continuous measure (0, 1, 2, 3 and 4+ medications) since 833 patients were taking 4 medications, and only 186 were taking more than 4 (ranging from 5 to 7). Given this small number, our continuous measure combines 5–7 medications into the 4+ group. In logistic regression, the Score test is asymptotically equivalent to the Cochran-Armitage trend test when the independent variable is continuous [32].

Multivariable associates of sociodemographic, fracture-related, and health-related characteristics were also evaluated using logistic regression. Low body weight was excluded because of a high level of missing data (nearly 20%). The chronic disease burden variable was also excluded due to potential collinearity with current medication count variable (Pearson correlation coefficient r = 0.87, *p* < .0001). The variable measuring the count of current medications was retained because it was deemed more important in the falls literature [19–22]. The size and statistical significance of the effects are reported as odds ratios (OR) and 95% confidence intervals (CI). All analyses were performed using SAS 9.4 [33].

## Results

From June 11, 2011 to November 5, 2014, 22,495 patients were screened and examined for eligibility by FPCs; of these, 21,980 were 50 years of age or older and had sustained a fragility fracture (confirmed eligible). In total, 21,869 patients were included in the study and analyzed because they had completed the baseline questionnaire. Patients included in the study who reported a language barrier (*n* = 1436) or mental/physical impairment (*n* = 1301) had a proxy complete the baseline questionnaire on their behalf, including all sections of the survey relevant to this study. The descriptive data for the whole sample and for the sample stratified by outcome status (screened at high falls risk) are presented in Table 1. Over one-quarter of the patients were 80 years of age or older, and 83.3% were female. Wrist fractures were the most commonly reported fracture type irrespective of whether or not patients were screened at high falls risk.

**Table 1:** Characteristics of fragility fracture patients stratified by outcome (screened at high falls risk)

Variable	Total, n (%)	Screened at high falls risk	
		Yes, n (%) ***n*** = 9735	No, n (%) ***n*** = 12,089
Age, years			
50–64	8112 (37.1)	2650 (27.2)	5455 (45.1)
65–79	8191 (37.5)	3523 (36.2)	4651 (38.5)
80+	5566 (25.5)	3562 (36.6)	1983 (16.4)
Sex			
Male	3663 (16.8)	1626 (16.7)	2030 (16.8)
Female	18,205 (83.3)	8109 (83.3)	10,058 (83.2)
Fracture location			
Hip	2838 (13.0)	1761 (18.1)	1064 (8.8)
Shoulder	3419 (15.6)	1614 (16.6)	1801 (14.9)
Wrist	8227 (37.6)	3077 (31.6)	5134 (42.5)
Ankle	3101 (14.2)	1204 (12.4)	1893 (15.7)
Other	4284 (19.6)	2079 (21.4)	2197 (18.2)
Living arrangement			
Alone in house or apartment	5591 (25.6)	2636 (27.1)	2951 (24.4)
With spouse, family member or roommate	14,059 (64.3)	5584 (57.4)	8453 (69.9)
Non-community dwelling	1486 (6.8)	1221 (12.5)	252 (2.1)
Missing, don’t know, refused	733 (3.4)	294 (3.0)	433 (3.6)
Alcohol use (more than 3/day)			
Yes	782 (3.6)	319 (3.3)	460 (3.8)
No	20,962 (95.9)	9355 (96.1)	11,565 (95.7)
Missing, don’t know, refused	125 (0.6)	61 (0.6)	64 (0.5)
Broke other bone since age 40 from a simple trip and fall			
Yes	6215 (28.4)	3625 (37.2)	2579 (21.3)
No	15,471 (70.7)	5982 (61.5)	9460 (78.3)
Missing, don’t know, refused	183 (0.8)	128 (1.3)	50 (0.4)
Taking Vitamin D supplements			
Yes	14,538 (66.5)	6555 (67.3)	7957 (65.8)
No	6903 (31.6)	2888 (29.7)	4003 (33.1)
Missing, don’t know, refused	428 (2.0)	292 (3.0)	129 (1.1)
Impairment or barrier			
Mental or physical	1301 (6.0)	1049 (10.8)	243 (2.0)
Language or other	1436 (6.6)	671 (6.9)	757 (6.3)
None	19,132 (87.5)	8015 (82.3)	11,089 (91.7)
Low body weight (< 60 kg)			
Yes	5166 (23.6)	2414 (24.8)	2746 (22.7)
No	12,505 (57.2)	5384 (55.3)	7106 (58.8)
Missing	4198 (19.2)	1937 (19.9)	2237 (18.5)
Chronic disease burden^a^			
3 or more conditions	4843 (22.2)	2919 (30.0)	1918 (15.9)
2 conditions	4258 (19.5)	2134 (21.9)	2117 (17.5)
1 condition	5329 (24.4)	2158 (22.2)	3161 (26.2)
No condition	5514 (25.2)	1538 (15.8)	3966 (32.8)
Missing	1925 (8.8)	986 (10.1)	927 (7.7)
Count of current medications^a^			
4 or more	1019 (4.7)	686 (7.1)	332 (2.8)
3	2379 (10.9)	1375 (14.1)	1000 (8.3)
2	3874 (17.7)	1973 (20.3)	1896 (15.7)
1	5384 (24.6)	2371 (24.4)	3003 (24.8)
0	7122 (32.6)	2270 (23.3)	4829 (40.0)
Missing, don’t know, refused	2091 (9.6)	1060 (10.9)	1019 (8.4)

^a^ For all chronic conditions including heart disease, diabetes, respiratory disease, cancer, arthritis, high blood pressure, and cholesterol

Overall, 9735 (44.5%) patients were categorized at high falls risk, and 12,089 (55.3%) were not; outcome data were missing for 45 patients. Among those screened at high falls risk, a much higher proportion were non-community dwelling (12.5%) compared to those who were not screened at high falls risk (2.1%). A mental or physical barrier was reported by 10.8% of patients screened at high falls risk, compared with 2.0% of those who were not. Similarly, current use of medications for chronic conditions was more commonly reported by patients screened at high falls risk. Specifically, 14.1 and 7.1% reported using 3 and 4+ medications, respectively. Among patients not screened at high falls risk, 8.3 and 2.8% reported using 3 and 4+ medications, respectively.

### Factors associated with being screened at high falls risk

Factors associated with being screened at high falls risk in unadjusted and multivariable (adjusted) logistic regression analyses are listed in Table 2. In unadjusted analyses, nearly all sociodemographic, fracture-related, and health-related characteristics were significantly associated with the outcome. The odds of being screened at high falls risk was 3.7 times higher for patients 80+ years of age compared to 50–64 year olds, and 7 times higher for those in non-community dwellings compared to patients living with a spouse, family member or roommate. A similarly strong association was observed for patients with a mental or physical barrier, whose odds of being screened at high falls risk was 6 times higher compared to patients with no reported impairments or barriers.

**Table 2:** Factors associated with being screened at high falls risk in unadjusted and multivariable (adjusted) logistic regression analyses

Variable	Unadjusted OR (95% CI)	Adjusted OR (95% CI)
Age, years		
50–64	1.00	1.00
65–79	1.56 (1.46–1.66)	1.14 (1.06–1.23)
80+	3.70 (3.44–3.97)	1.72 (1.57–1.88)
Sex		
Male	1.00	1.00
Female	1.01 (0.94–1.08)	1.06 (0.97–1.15)
Fracture location		
Hip	2.76 (2.53–3.02)	1.75 (1.58–1.95)
Shoulder	1.50 (1.38–1.62)	1.26 (1.15–1.39)
Wrist	1.00	1.00
Ankle	1.06 (0.98–1.16)	1.18 (1.07–1.29)
Other	1.58 (1.47–1.70)	1.35 (1.24–1.47)
Living arrangement		
Alone in house or apartment	1.35 (1.27–1.44)	1.20 (1.11–1.28)
With spouse, family member or roommate	1.00	1.00
Non-community dwelling	7.33 (6.38–8.43)	3.42 (2.87–4.07)
Alcohol use (more than 3/day)		
Yes	0.86 (0.74–0.99)	0.99 (0.84–1.17)
No	1.00	1.00
Broke other bone since age 40 from a simple trip and fall		
Yes	2.22 (2.09–2.36)	1.88 (1.75–2.01)
No	1.00	1.00
Taking Vitamin D supplements		
Yes	1.14 (1.08–1.21)	0.97 (0.90–1.03)
No	1.00	1.00
Impairment or barrier		
Mental or physical	5.97 (5.18–6.89)	2.51 (2.09–3.01)
Language/other	1.23 (1.10–1.37)	0.97 (0.85–1.11)
None	1.00	1.00
Low body weight (< 60 kg)		
Yes	1.16 (1.09–1.24)	–
No	1.00	
Chronic disease burden^a^		
3 or more conditions	3.92 (3.61–4.26)	–
2 conditions	2.60 (2.39–2.83)	
1 condition	1.76 (1.62–1.91)	
No condition	1.00	
Count of current medications^a^		
4 or more	4.40 (3.83–5.07)	3.42 (2.95–3.97)
3	2.93 (2.66–3.22)	2.28 (2.05–2.53)
2	2.22 (2.05–2.40)	1.72 (1.57–1.88)
1	1.68 (1.56–1.81)	1.37 (1.26–1.48)
0	1.00	1.00

^a^ For all chronic conditions including heart disease, diabetes, respiratory disease, cancer, arthritis, high blood pressure, and cholesterol

In terms of current use of medications for chronic conditions, taking a greater number of medications was strongly associated with being screened at high falls risk (Table 2). This was further evaluated using a trend test; specifically, the Score test was significant (*p* < .0001), and we found an OR of 1.44 (95% CI: 1.40–1.47) (results not shown). This indicates that for every increase of one in the number of medications used, there was a 44% higher odds of being screened at high falls risk.

In the multivariable logistic regression model containing all factors of interest, we found that most variables had similar effects as in unadjusted analyses and retained statistical significance, except for alcohol use and taking vitamin D supplements. We observed a reduction in the strength of associations between some key factors and the outcome; however, these variables were still strongly associated with the outcome. Specifically, being 80+ years of age (vs. 50–64 years of age), non-community dwelling (vs. living with spouse, family member or roommate), having a mental or physical impairment (vs. none), and taking multiple medications, all remained strongly associated with being screened at high falls risk. For the latter variable, there was a statistically significant association where patients taking more medications had a higher odds of being screened at high falls risk (Table 2).

## Discussion

As part of our large-scale provincial fracture screening program, using an adapted tool (STEADI) to screen for fall risk revealed that nearly 45% of our population of fragility fracture patients were screened at high falls risk. A cohort study in the United States reported nearly 38% of patients with osteoporosis were at high fall risk based on an adapted version of the STEADI algorithm [34], but we have not found a similar study focused solely on fragility fracture patients. Other studies have reported fall risk using STEADI or STEADI-related measures of fall risk in older populations, with findings ranging from 21.3% [35] to 35% at a high risk of falls [36]. However, the authors of these other studies did not indicate whether or not study participants had sustained fragility fractures.

Our results confirm that being 80+ years of age (vs. 50–64 years) is related to being screened at high falls risk. The role of advanced age in falls is a well-established finding in the literature [2, 13, 14]. However, the strength of the association between older age and being screened at high falls risk in our data was lessened in multivariable analyses. This is possibly due to the effects of fracture location in the model, specifically hip fractures, which are known to be more common amongst older individuals and be associated with subsequent falls [37, 38]. One of our unexpected findings was that sex was not statistically significantly associated with being screened at high falls risk. This is consistent with Smee et al. [39] who found that sex was not related to fall risk in community-living older adults. Similarly, Dewan et al. [1] reported that sex was not associated with subsequent falls or fragility fractures in their cohort of older patients with distal radius fracture. This was however contradictory to previous studies which reported that women were at greater risk for falls and fall injuries [2, 15].

The odds of screening at high falls risk was nearly 3.5 times higher for patients in non-community dwellings (vs. those living with a spouse, family member or roommate), even when controlling for other variables (e.g., mental or physical impairment). Rubenstein [7] reported that persons living in long-term care institutions have much higher rates of falls, and that 10–25% of such falls result in serious injuries (e.g., fracture or laceration). Furthermore, Todd and Skelton [22] reported that nursing home residents with diagnosed dementia fell twice as often as those with normal cognition. While not examined among nursing home residents specifically, our results indicate that the odds of screening at high falls risk was over 2.5 times higher in patients with mental or physical impairment compared to those without any impairment. This strong association remained even after controlling for related factors such as age, living arrangement, and medication use. Not surprisingly, cognitive impairment is strongly associated with an increased risk of falling in the literature [3, 20, 22].

Finally, we found that there was a strong association between taking more medications for chronic conditions and being screened at high falls risk. When compared to patients not taking any medications, the odds of screening at high falls risk was 3.4 times higher for those taking 4+ medications in adjusted analyses. Others have similarly found that the risk is increased significantly if a person is on more than four medications, irrespective of type [19–22]. Indeed, a review by Zia et al. [40] concluded that polypharmacy was a significant factor in the risk of falls, regardless of whether it was defined as ≥4 or ≥ 5 medications, or whether or not fall-risk-increasing drugs were used. Furthermore, Helgadóttir et al. [17] found that an increasing number of medications increased risk of fall injury in a population-based study of Swedish people 65 years and older. However, the association was partly explained by age and sex, and there was no clear dose-response relationship.

## Strengths and limitations

### Strengths

This study investigated the factors associated with being screened at high falls risk in fragility fracture patients using a large, provincial database. This was an important strength as our study was based on a multicenter cohort of individuals representing the fragility fracture population in Ontario. Our work addresses a gap in the literature by focusing on participants with prevalent fragility fractures who screened positive for high falls risk. Another strength is our outcome measure, which was adapted from a validated and commonly used tool (STEADI) to measure fall risk [34, 41, 42].

### Limitations

A limitation of this study was that our analysis was cross-sectional. We therefore cannot make any statements regarding causality or in terms of predicting the subsequent risk of falling in our fragility fracture population. However, this may be an area for future research as our provincial program is in the process of incorporating actual fall events in the follow-up questionnaires administered as part of the FSPP. This could allow us to examine the prevalence of falls at follow-up and to assess risk factors predicting subsequent falls in fragility fracture patients. Furthermore, the variable measuring impairment or barrier combines mental and physical impairment into one category, because this is how the question was asked in the questionnaire. This variable was meant to be a proxy for impaired cognition; however, we do not know the extent to which patients in this group actually had a mental impairment, or even the type of mental impairment. Nonetheless, we found the strong association between mental or physical impairment and being screened at high falls risk was comparable to other studies, providing some evidence that our variable was a good proxy for cognitive impairment. Finally, participants had to recall if they had fallen in the past year, besides the fall that may have led to the current fracture(s). This type of information is subject to recall bias [43, 44], which can lead to underestimation of risk. However, as indicated in their evidence review and recommendation statements, the US Preventive Services Task Force found that history of falls was the most commonly used item in risk assessment tools, and it consistently identified persons at high risk for falls [45].

## Conclusions

We found that a large proportion (nearly 45%) of prevalent fragility fracture patients in a provincial multicentre cohort was identified as being screened at high falls risk. Living in a non-community dwelling, and taking 4 or more medications were the strongest independent variables associated with being screened at high falls risk. These are potentially modifiable characteristics that should be considered when assessing falls risk in fragility fracture patients, and particularly when designing interventions for preventing subsequent falls in this population. These may include regular medication reviews, such as withdrawal of cardiovascular medications [40]. Although there is little evidence of a reduction in the risk of falls with the withdrawal or dose reduction of fall-risk-increasing medication [40], prescriptions for older patients “should be individualized and subject to frequent periodic reviews, while consistently striving to minimize the total number and amount of medications consumed by thepatient” [40]. Interventions that address the social environment for those in nursing homes should also be considered; e.g., staff ratio and staff training [14]. Ongoing work to address the higher risk of falls in the fragility fracture population is warranted, with a particular eye to addressing the needs of more vulnerable patients.

## Data Availability

The data that support the findings of this study are available from Osteoporosis Canada but restrictions apply to the availability of these data, which were used under license for the current study, and so are not publicly available. Data are however available from the authors upon reasonable request and with permission of Osteoporosis Canada.

## Abbreviations

FPCs: Fracture Prevention Coordinators
FSPP: Fracture Screening and Prevention Program
STEADI: Stopping Elderly Accidents, Deaths & Injuries
OR: Odds ratio
CI: Confidence interval

## References

[1] Dewan N, MacDermid J, Grewal R, Beattie K (2018). Risk factors predicting subsequent falls and osteoporotic fractures at 4 years after distal radius fracture-a prospective cohort study. Arch Osteoporos.

[2] Chang V, Do M (2015). Risk factors for falls among seniors: implications of gender. Am J Epidemiol.

[3] Ambrose A, Cruz L, Paul G (2015). Falls and fractures: a systematic approach to screening and prevention. Maturitas..

[4] Tinetti ME, Gordon C, Sogolow E, Lapin P, Bradley EH (2006). Fall-risk evaluation and management: challenges in adopting geriatric care practices. Gerontologist..

[5] National Institute for Health and Clinical Excellence. Falls in older people: assessing risk and prevention [CG161]. 2013. https://www.nice.org.uk/guidance/cg161/chapter/1-recommendations. Accessed 01 Apr 2019. 31869045

[6] Smulders E, Van LW LR, Duysens J, Weerdesteyn V (2011). Does osteoporosis predispose falls? A study on obstacle avoidance and balance confidence. BMC Musculoskelet Disord.

[7] Rubenstein LZ (2006). Falls in older people: epidemiology, risk factors and strategies for prevention. Age Ageing.

[8] Einsiedel T, Becker C, Stengel D, Schmelz A, Kramer M, Daxle M, Lechner F, Kinzl L, Gebhard F (2006). Do injuries of the upper extremity in geriatric patients end up in helplessness? A prospective study for the outcome of distal radius and proximal humerus fractures in individuals over 65. Z Gerontol Geriatr.

[9] Papaioannou A, Morin S, Cheung AM, Atkinson S, Brown JP, Feldman S, Hanley DA, Hodsman A, Jamal SA, Kaiser SM, Kvern B, Siminoski K, Leslie WD (2010). 2010 clinical practice guidelines for the diagnosis and management of osteoporosis in Canada: summary. CMAJ..

[10] Edwards BJ, Song J, Dunlop DD, Fink HA, Cauley JA (2010). Functional decline after incident wrist fractures--study of osteoporotic fractures: prospective cohort study. BMJ..

[11] Russell MA, Hill KD, Blackberry I, Day LL, Dharmage SC (2006). Falls risk and functional decline in older fallers discharged directly from emergency departments. J Gerontol A Biol Sci Med Sci.

[12] Bliuc D, Nguyen ND, Milch VE, Nguyen TV, Eisman JA, Center JR (2009). Mortality risk associated with low-trauma osteoporotic fracture and subsequent fracture in men and women. JAMA..

[13] Deandrea S, Lucenteforte E, Bravi F, Foschi R, La VC, Negri E (2010). Risk factors for falls in community-dwelling older people: a systematic review and meta-analysis. Epidemiol..

[14] Hopewell S, Adedire O, Copsey BJ, Sherrington C, Clemson LM, Close JC, Lamb SE. Multifactorial and multiple component interventions for preventing falls in older people living in the community. Cochrane Database Syst Rev. 2018. 10.1002/14651858.CD012221.pub2. 10.1002/14651858.CD012221.pub2 PMC6513234 30035305

[15] Stevens JA, Ballesteros MF, Mack KA, Rudd RA, DeCaro E, Adler G (2012). Gender differences in seeking care for falls in the aged Medicare population. Am J Prev Med.

[16] Lawlor DA, Patel R, Ebrahim S (2003). Association between falls in elderly women and chronic diseases and drug use: cross sectional study. BMJ..

[17] Helgadottir B, Laflamme L, Monarrez-Espino J, Moller J (2014). Medication and fall injury in the elderly population; do individual demographics, health status and lifestyle matter?. BMC Geriatr.

[18] Munson JC, Bynum JP, Bell JE, Cantu R, McDonough C, Wang Q, Tosteson TD, Tosteson AN (2016). Patterns of prescription drug use before and after fragility fracture. JAMA Intern Med.

[19] Freeland KN, Thompson AN, Zhao Y, Leal JE, Mauldin PD, Moran WP (2012). Medication use and associated risk of falling in a geriatric outpatient population. Ann Pharmacother.

[20] Bergland A, Wyller TB (2004). Risk factors for serious fall related injury in elderly women living at home. Inj Prev.

[21] Hartikainen S, Lonnroos E, Louhivuori K (2007). Medication as a risk factor for falls: critical systematic review. J Gerontol A Biol Sci Med Sci.

[22] Todd C , Skelton D. What are the main risk factors for falls among older people and what are the most effective interventions to prevent these falls? World Health Organization. 2004. http://www.euro.who.int/document/E82552.pdf. Accessed 22 Mar 2019.

[23] Campbell AJ, Robertson MC (2006). Implementation of multifactorial interventions for fall and fracture prevention. Age Ageing.

[24] McClure RJ, Turner C, Peel N, Spinks A, Eakin E, Hughes K. Population-based interventions for the prevention of fallrelated injuries in older people. Cochrane Database Syst Rev. 2005. 10.1002/14651858.CD004441.pub2. 10.1002/14651858.CD004441.pub2 PMC11802279 15674948

[25] Menant JC, Close JC, Delbaere K, Sturnieks DL, Trollor J, Sachdev PS, Brodaty H, Lord SR (2012). Relationships between serum vitamin D levels, neuromuscular and neuropsychological function and falls in older men and women. Osteoporos Int.

[26] Gillespie LD, Robertson MC, Gillespie WJ, Sherrington C, Gates S, Clemson LM, Lamb SE. Interventions for preventing falls in older people living in the community. Cochrane Database Syst Rev. 2012. 10.1002/14651858.CD007146.pub3. 10.1002/14651858.CD007146.pub3 PMC8095069 22972103

[27] Pluijm SM, Smit JH, Tromp EA, Stel VS, Deeg DJ, Bouter LM, Lips P (2006). A risk profile for identifying community-dwelling elderly with a high risk of recurrent falling: results of a 3-year prospective study. Osteoporos Int.

[28] Jaglal SB, Hawker G, Cameron C, Canavan J, Beaton D, Bogoch E, Jain R, Papaioannou A (2010). The Ontario osteoporosis strategy: implementation of a population-based osteoporosis action plan in Canada. Osteoporos Int.

[29] Kannus P, Sievanen H, Palvanen M, Jarvinen T, Parkkari J (2005). Prevention of falls and consequent injuries in elderly people. Lancet..

[30] Bessette L, Ste-Marie LG, Jean S, Davison KS, Beaulieu M, Baranci M, Bessant J, Brown JP (2008). The care gap in diagnosis and treatment of women with a fragility fracture. Osteoporos Int.

[31] Centers for Disease Control and Prevention. STEADI - Older Adult Fall Prevention. Centers for Disease Control and Prevention, National Center for Injury Prevention and Control. 2019. https://www.cdc.gov/steadi/index.html. Accessed 05 Apr 2019.

[32] Liu H. Cochran-Armitage trend test using SAS. Merck & Co., Inc. 2007. https://www.lexjansen.com/pharmasug/2007/sp/SP05.pdf. Accessed 29 Mar 2019.

[33] SAS software, Version 9.4. Copyright © 2013. Cary; SAS Institute Inc.; 2013.

[34] Lohman MC, Crow RS, DiMilia PR, Nicklett EJ, Bruce ML, Batsis JA (2017). Operationalisation and validation of the stopping elderly accidents, deaths, and injuries (STEADI) fall risk algorithm in a nationally representative sample. J Epidemiol Community Health.

[35] Albert SM, King J, Boudreau R, Prasad T, Lin CJ, Newman AB (2014). Primary prevention of falls: effectiveness of a statewide program. Am J Public Health.

[36] Casey CM, Parker EM, Winkler G, Liu X, Lambert GH, Eckstrom E (2016). Lessons learned from implementing CDC's STEADI falls prevention algorithm in primary care. Gerontologist..

[37] Lloyd BD, Williamson DA, Singh NA, Hansen RD, Diamond TH, Finnegan TP, Allen BJ, Grady JN, Stavrinos TM, Smith EU, Diwan AD, Fiatarone Singh MA (2009). Recurrent and injurious falls in the year following hip fracture: a prospective study of incidence and risk factors from the sarcopenia and hip fracture study. J Gerontol A Biol Sci Med Sci.

[38] Shumway-Cook A, Ciol MA, Gruber W, Robinson C (2005). Incidence of and risk factors for falls following hip fracture in community-dwelling older adults. Phys Ther.

[39] Smee DJ, Anson JM, Waddington GS, Berry HL. Association between physical functionality and falls risk in community-living older adults. Curr Gerontol Geriatr Res. 2012. 10.1155/2012/864516. 10.1155/2012/864516 PMC3529454 23304137

[40] Zia A, Kamaruzzaman SB, Tan MP (2015). Polypharmacy and falls in older people: balancing evidence-based medicine against falls risk. Postgrad Med.

[41] Stevens JA, Phelan EA (2013). Development of STEADI: a fall prevention resource for health care providers. Health Promot Pract.

[42] Stevens JA (2013). The STEADI tool kit: a fall prevention resource for health care providers. IHS Prim Care Provid.

[43] Garcia PA, Dias JMD, Silva SLA, Dias RC (2015). Prospective monitoring and self-report of previous falls among older women at high risk of falls and fractures: a study of comparison and agreement. Braz J Phys Ther.

[44] Lawson SN, Zaluski N, Petrie A, Arnold C, Basran J, Bello-Haas VD (2013). Validation of the Saskatoon falls prevention Consortium's falls screening and referral algorithm. Physiother Can.

[45] Grossman DC, Curry SJ, Owens DK, Barry MJ, Caughey AB, Davidson KW, Doubeni CA, Epling JW, Kemper AR, Krist AH, Kubik M, Landefeld S, Mangione CM, Pignone M, Silverstein M, Simon MA, Tseng CW (2018). Interventions to prevent falls in community-dwelling older adults: US preventive services task force recommendation statement. JAMA..

